# Low Prevalence of Nirmatrelvir-Ritonavir Resistance-Associated Mutations in SARS-CoV-2 Lineages From Botswana

**DOI:** 10.1093/ofid/ofae344

**Published:** 2024-07-02

**Authors:** Wonderful T Choga, Ontlametse T Bareng, Natasha O Moraka, Dorcas Maruapula, Irene Gobe, Nokuthula S Ndlovu, Boitumelo J L Zuze, Patience C Motshosi, Kedumetse B Seru, Teko Matsuru, Matshwenyego Boitswarelo, Mogomotsi Matshaba, Tendani Gaolathe, Mosepele Mosepele, Joseph Makhema, Trevor J M Tamura, Jonathan Z Li, Roger Shapiro, Shahin Lockman, Simani Gaseitsiwe, Sikhulile Moyo

**Affiliations:** Botswana Harvard Health Partnership, Gaborone, Botswana; School of Applied Health Sciences, Faculty of Health Sciences, University of Botswana, Gaborone, Botswana; Botswana Harvard Health Partnership, Gaborone, Botswana; School of Applied Health Sciences, Faculty of Health Sciences, University of Botswana, Gaborone, Botswana; Botswana Harvard Health Partnership, Gaborone, Botswana; School of Applied Health Sciences, Faculty of Health Sciences, University of Botswana, Gaborone, Botswana; Botswana Harvard Health Partnership, Gaborone, Botswana; School of Applied Health Sciences, Faculty of Health Sciences, University of Botswana, Gaborone, Botswana; Botswana Harvard Health Partnership, Gaborone, Botswana; Department of Biological Sciences and Biotechnology, Faculty of Sciences, Botswana International University of Science and Technology, Palapye, Botswana; Botswana Harvard Health Partnership, Gaborone, Botswana; Botswana Harvard Health Partnership, Gaborone, Botswana; Botswana Harvard Health Partnership, Gaborone, Botswana; Botswana Harvard Health Partnership, Gaborone, Botswana; Department of Health Systems Management, Clinical Services, Ministry of Health Botswana, Gaborone, Botswana; Botswana-Baylor Children's Clinical Centre of Excellence, Gaborone, Botswana; Department of Pediatrics, Baylor College of Medicine, Houston, Texas, USA; Botswana Harvard Health Partnership, Gaborone, Botswana; Department of Internal Medicine, Faculty of Medicine, University of Botswana, Gaborone, Botswana; Botswana Harvard Health Partnership, Gaborone, Botswana; Department of Internal Medicine, Faculty of Medicine, University of Botswana, Gaborone, Botswana; Botswana Harvard Health Partnership, Gaborone, Botswana; Department of Immunology and Infectious Diseases, Harvard T.H. Chan School of Public Health, Boston, Massachusetts, USA; Division of Infectious Diseases, Brigham & Women's Hospital, Boston, Massachusetts, USA; Division of Infectious Diseases, Brigham & Women's Hospital, Boston, Massachusetts, USA; Botswana Harvard Health Partnership, Gaborone, Botswana; Department of Immunology and Infectious Diseases, Harvard T.H. Chan School of Public Health, Boston, Massachusetts, USA; Botswana Harvard Health Partnership, Gaborone, Botswana; Department of Immunology and Infectious Diseases, Harvard T.H. Chan School of Public Health, Boston, Massachusetts, USA; Harvard Medical School, Boston, Massachusetts, USA; Botswana Harvard Health Partnership, Gaborone, Botswana; Department of Immunology and Infectious Diseases, Harvard T.H. Chan School of Public Health, Boston, Massachusetts, USA; Botswana Harvard Health Partnership, Gaborone, Botswana; School of Applied Health Sciences, Faculty of Health Sciences, University of Botswana, Gaborone, Botswana; Department of Immunology and Infectious Diseases, Harvard T.H. Chan School of Public Health, Boston, Massachusetts, USA; School of Health Systems and Public Health, University of Pretoria, Pretoria, South Africa; Division of Medical Virology, Faculty of Medicine and Health Sciences, Stellenbosch University, Cape Town, South Africa

**Keywords:** Botswana, nirmatrelvir-ritonavir, Paxlovid, resistance mutations, SARS-CoV-2

## Abstract

**Background:**

We evaluated naturally occurring nirmatrelvir-ritonavir (NTV/r) resistance-associated mutations (RAMs) among severe acute respiratory syndrome coronavirus 2 (SARS-CoV-2) strains from Botswana, a country with no NTV/r use to date, in order to recommend the usage of the agent for high-risk patients with coronavirus disease 2019 (COVID-19).

**Methods:**

We conducted a retrospective analysis using 5254 complete SARS-CoV-2 sequences from Botswana (September 2020–September 2023). We evaluated the mutational landscape of SARS-CoV-2 3-Chymotrypsin-like protease (3CLpro) relative to the highlighted list of RAMs granted Food and Drug Administration Emergency Use Authorization in 2023.

**Results:**

The sequenced 5254 samples included Beta variants of concerns (VOCs; n = 323), Delta VOCs (n = 1314), and Omicron VOCs (n = 3354). Overall, 77.8% of the sequences exhibited at least 1 polymorphism within 76/306 amino acid positions in the nsp5 gene. NTV/rRAMs were identified in 34/5254 (0.65%; 95% CI, 0.43%–0.87%) and occurred at 5 distinct positions. Among the NTV/r RAMS detected, A191V was the most prevalent (24/34; 70.6%). Notably, T21I mutation had a prevalence of 20.6% (7/34) and coexisted with either K90R (n = 3) polymorphism in Beta sequences with RAMs or P132H (n = 3) polymorphism for Omicron sequences with RAMs. Other NTV/r RAMs detected included P108S, with a prevalence of 5.88% (2/34), and L50F, with a prevalence of 2.94% (1/34). NTV/r RAMs were significantly higher (*P* < .001) in Delta (24/35) compared with Beta (4/34) and Omicron (6/34) sequences.

**Conclusions:**

The frequency of NTV/r RAMs in Botswana was low. Higher rates were observed in Delta VOCs compared to Omicron and Beta VOCs. As NTV/r use expands globally, continuous surveillance for drug-resistant variants is essential, given the RAMs identified in our study.

## Teaser

We characterised nirmatrelvir-ritonavir (NTV/r) resistance-associated mutations (RAMs) in SARS-CoV-2 genomes from Botswana (2020-2023), a country with no NTV/r use. RAMs frequency (0.65%: 95%CI:0.43-0.87) was comparable to the global estimate (0.5%). We observed 10 deleterious Mpro-mutations that may impact NTV/r use.

Severe acute respiratory syndrome coronavirus 2 (SARS-CoV-2) is the primary cause of coronavirus disease 2019 (COVID-19), a global epidemic with substantial risks [1–4]. Antiviral therapy for SARS-CoV-2 such as nirmatrelvir-ritonavir (NTV/r) has made COVID-19 more manageable, reducing deaths and hospitalization rates [5–10]. Even though NTV/r is a highly potent antiviral that has shown low rates of virological failure, the development of resistant-associated mutations (RAMs) poses a threat to its effectiveness [11]. As SARS-CoV-2 is an RNA virus, mutations of concern associated with resistance to antivirals may naturally develop in a treatment-naïve population due to errors introduced into the genome during replication.

During SARS-CoV-2 replication, the biggest polyprotein 1ab (PP1ab) is generated from ORF1ab for Coronaviridae [12]. The polyprotein undergoes cleavage by the viral proteases papain-like protease (PL^pro^ or nsp3) and homodimeric cysteine 3-Chymotrypsin-like protease (3CL^pro^, also known as M^pro^ or nonstructural protein 5 [nsp5]) at multiple junctions to generate a series of nonstructural proteins (nsp1-16), which form the replicase machinery essential to the virus life cycle [13, 14].

NTV/r was approved under Emergency Use Authorization (EUA) by the Food and Drug Administration (FDA) in December of 2021 as the first orally bioavailable SARS-CoV-2 M^pro^ inhibitor for the treatment of individuals infected with SARS-CoV-2 and deemed at a high risk of developing severe illness [7]. Development of mutations across 3CL^pro^ active site residues directly impacts substrate adherence and drug resistance. The NTV/r resistance hotspots include the M165T, S144, H172F/Q/Y, M165T, E166N/V, Q189E/K, and Q192S/T/V mutants, which have been associated with reduced NTV inhibitory activity of SARS-CoV-2 replication or 3CL^pro^ activity [15]. While NTV/r is unaffected by rapid SARS-CoV-2 spike evolution, the ongoing emergence of new SARS-CoV-2 variants, some with mutations in ORF1a, highlights mutational adaptability, signals potential persistence of the pandemic, and raises concerns about the reduced efficacy of antiviral treatments due to drug-resistant variants. Hence, it remains crucial to assess the baseline RAM landscape for any candidate drugs to ascertain efficacy.

Several studies have evaluated the safety, effectiveness, and emergency resistance mutations of NTV/r in different settings and populations [7, 16–20]; however, there are limited data on M^pro^ resistance mutations in treatment-naïve populations.

Botswana reported 330 215 SARS-CoV-2 cases and 2800 deaths as of August 2023 [21]. Botswana has implemented the COVID-19 test and treatment national program, and NTV/r could be a treatment option for high-risk people who are infected with SARS-CoV-2. Such interventions within low- to middle-income countries (LMICs) are highly desirable as patients can be treated as outpatients without overburdening the health care system. Similarly, the country has successfully implemented a near real-time national SARS-CoV-2 genomic surveillance system covering all 9 COVID-19 zones in the country, offering a unique opportunity to assess potential NTV/r RAMs and escape mutations. Therefore, we sought to characterize the baseline RAM landscape of M^pro^ in all the SARS-CoV-2 sequences generated in Botswana. This first report provides insights into the use of NTV/r for treating COVID-19 patients in our setting. Botswana was equally affected by different SARS-CoV-2 variants and subvariants and had reported high cases of COVID-19; therefore, the study will provide insight on the possibility of NTV/r use in any setting with low or high numbers of SARS-CoV-2 cases, especially Sub-Saharan African countries and neighboring countries.

## METHODS

### Study Design, Sample Collection, and Selection

This was a retrospective analysis of SARS-CoV-2 sequences previously generated during routine national diagnostic testing, surveillance, and sequencing in Botswana. For routine and clinical COVID-19 diagnosis, combined nasopharyngeal and oropharyngeal (N/O) samples were collected. The N/O samples were routinely and randomly sampled per week between September 2020 and September 2023 from all regions of the 9 nationwide COVID-19 zones and sent to the nearest laboratory with COVID-19 polymerase chain reaction (PCR) method testing capacity. SARS-CoV-2 sequencing and analysis were conducted at Botswana Harvard HIV Reference Laboratory (BHHRL), which serves as the main sequencing hub for SARS-CoV-2 genomic surveillance in Botswana. The confirmed SARS-CoV-2 residual samples from individuals who tested positive for COVID-19 were randomly and routinely sampled weekly from all 9 COVID-19 zones in Botswana for genomic surveillance. Sample selection was often random; otherwise, convenient sampling was employed if the number of confirmed weekly cases was modest in each location. The selected samples did not necessarily represent the proportion of cases in each zone. In certain instances, such as outbreaks—for example, schools—we sequenced all the available samples. Preferably, all samples with a real-time cycle threshold value <35 (qCt ≤35) were selected. Throughout the sample collection period (September 2020–September 2023), Botswana had not incorporated SARS-CoV-2 antiretroviral therapies (ARTs) into its national guidelines. However, NTV/r was still being procured and not yet in use.

### Library Preparation and Next Next-Generation Sequencing

Reverse transcription quantitative PCR (RT-qPCR) was performed for SARS-CoV-2 diagnostics using the 2019-nCoV RNA (PCR-Fluorescence Probing) Assay (Sun Yat-sen University, Da An Gene Co., Ltd, China) according to the manufacturer's instructions. Before June 2021, sequences were prepared using the Native Barcoding Kit (EXP-NBD196) in conjunction with the ARTIC nCoV-2019 V3 sequencing protocol, which generated 400-bp tiled amplicons [22, 23]; after June 2021, we used midnight protocol to generate complete genomes based on 1200-bp tiled amplicons [24]. In each of the sequencing batches, a nontemplate control was included across all steps to check for any cross-contamination. Nanopore sequencing was performed using Oxford Nanopore Technologies (Oxford, UK; MinION, Mk1B, Mk1C, and GridION). Depending on the period of sequencing, we used the latest available MinKNOW release version for demultiplexing and high-accuracy base-calling.

### Next-Generation Sequence Analysis and SARS-CoV-2 Lineage Classification

The raw FASTQ sequence output files were processed into consensus FASTA files, followed by reference-based assembly using the ARTIC nCoV-2019 novel coronavirus bioinformatics protocol and Genome Detective [25, 26]. To assess the quality control reports, NextClade [27] was used, and the resulting consensus sequences were further manually imputed in AliView [28]. This was done to correct indels that may arise due to sequencing errors and cause ORF shifts. Any non-ATGC characters or STOP codons were replaced with a triplet of hyphens, including converting the partial indel to an indel (eg, TC- to —). Consensus FASTA files obtained were assessed for quality (eg, presence of private mutations, >80% coverage) and assigned to clades and lineages using the latest version of NextClade [27]. The final consensus edited sequences and associated metadata were deposited. All sequences that met the threshold were deposited in the Global Initiative on Sharing All Influenza Data (GISAID; https://doi.org/10.55876/gis8.240523kh).

### Genetic and Mutational Profiling of SARS-CoV-2 M^pro^

We analyzed genome sequences and patient metadata for 5254 isolates from Botswana. The 5254 sequences were from individual patients who were presumed NTV/r-naïve. All the sequences were aligned using NextClade and NC_045512.2 (Wuhan-Hu-1) reference annotations. M^pro^ nucleotide sequences (ORF1ab: 13 441–16 236) were extracted from the MSA using NextClade [27]. An ad hoc script was used to curate mutations occurring on the M^pro^, stratified by lineages. The mutation density index was calculated by dividing the prevalence of mutations by the total number of sequences for that lineage. Mutations were subsequently merged into their metadata (age, sex, sample location, lineage, and collection date). NTV/r RAMs were assessed a priori using published data including the highlighted list of mutations from the 2023 EUA and those described in the literature (Supplementary Table 1) [29–34].

### Evaluating in Silico the Potential Impact of Emerging M^pro^ Polymorphisms

SNAP2 [35, 36], Phyre [37], and PolyPhen [38] were used to predict in silico the potential impact (deleterious or neutral) and nature of mutations located within the M^pro^ region of SARS-CoV-2. The algorithm calibration was performed as previously described [39, 40]. Subsequently, we validated the results by assessing the accuracy of predictions for known deleterious mutations reported in the literature. To characterize potentially new mutations of concern, our focus was on rare mutations (occurring in <10 per million sequences) that had not been previously reported. The Phyre2 [38] tool was used to reconstruct the 3D structures of the M^pro^ gene using protein homology modeling, and PyMOL 3 [41] was used for visual inspection and annotating deleterious positions.

### Statistical Analysis

Characteristics of the individuals were described using proportions with 95% CIs and medians with interquartile ranges (IQRs). The Kruskal-Wallis test was used for comparisons of continuous variables between variants of concern (VOCs) with NTV/r RAMs. We reported categorical variables as counts and percentages and conducted comparisons using the Pearson chi-square test or Fisher exact test, as appropriate. A 2-sided *P* < .05 was considered statistically significant. The prevalence of mutations was estimated with 95% CIs using the binomial exact method for each group. The prevalence of RAMs was (i) estimated for overall sequences and (ii) stratified by VOCs. The prevalence of mutations among VOCs was compared using a comparison of proportions test. A data analysis was conducted using R statistical software, version 4.2.2 (R Core Team, R Foundation for Statistical Computing, Vienna, Austria), and Stata 16.1 (StataCorp, College Station, TX, USA).

### Phylogenetic Analysis

Briefly, all sequences with NTV/r RAMs were stratified based on lineage assignment, and then codon alignments were constructed using NextAlign [27]. A maximum-likelihood (ML) tree was inferred from the resulting alignment in IQ-TREE 2 [42] using the General Time Reversible (GTR) model of nucleotide substitution with empirical base frequencies with gamma distributed over site-to-site variations in nucleotide substitution rates, as determined by jModelTest2 [43]. Statistical supports for nodes of the ML phylogeny were assessed using a bootstrap approach with 1000 replicates. The reliability of the observed clusters was established based on internal node bootstrap values exceeding 80%.

### Patient Consent

This retrospective analysis was conducted as part of SARS-CoV-2 genomic surveillance generated during routine diagnostic testing and sequencing, which was approved by the Health Research and Development Committee (Protocol #HRDC00945; HPDME 13/18/1), the Harvard T.H. Chan School of Public Health Office of Research Administration (Protocol #IRB21-1661), and the Mass General Brigham Institutional Review Board (Protocol #2022P00421). This retrospective analysis was conducted as part of SARS-CoV-2 genomic surveillance generated using residual samples from routine diagnostic testing and sequencing, which was approved by the Health Research and Development Committee (Protocol #HRDC00945; HPDME 13/18/1), the Harvard T.H Chan School of Public Health Office of Research Administration (Protocol #IRB21-1661), and the Mass General Brigham Institutional Review Board (Protocol #2022P00421). Waivers of consent were obtained from the institutional review boards. The study was carried out in accordance with the Helsinki Declaration's guiding principles and local institutional review board guidelines.

## RESULTS

### Prevalence of Nirmatrelvir-Ritonavir RAMs

**Figure 1.**
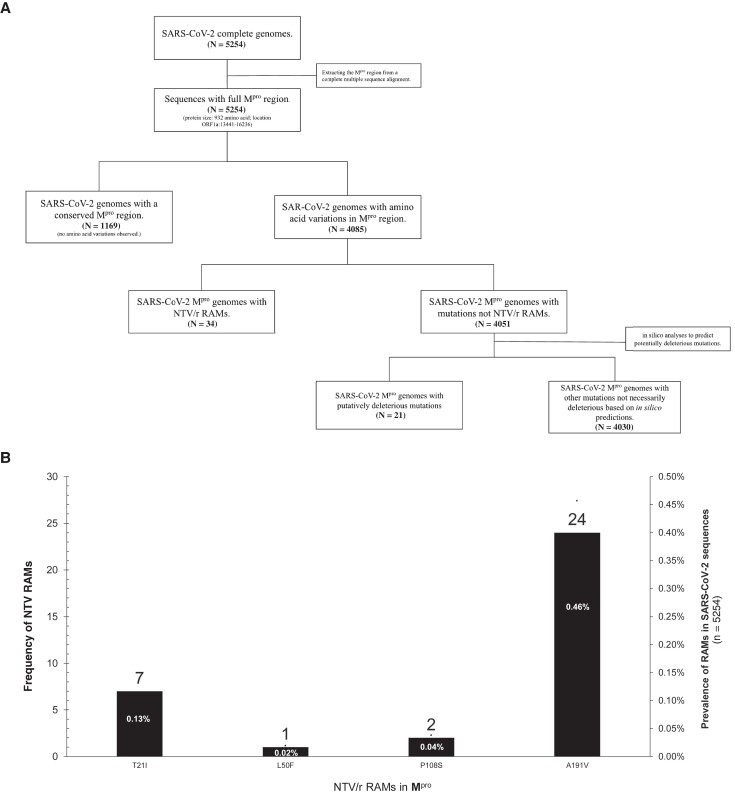
A, Flowchart showing the distribution of complete SARS-CoV-2 sequences (n = 5254) generated in the study. B, Distribution of major NTV/r RAMs in the ns5p gene. Abbreviations: NTV/r, nirmatrelvir-ritonavir; RAMs, resistance-associated mutations; SARS-CoV-2, severe acute respiratory syndrome coronavirus 2.

NTV/r RAMs were identified in 34 out of 5254 samples, giving an overall prevalence of 0.65% (95% CI, 0.43%–0.87%) (Table 1). None of the 34 individuals with NTV/r RAMs had received NTV/r (Figure 1A). The median age of individuals with NTV/r RAMs (IQR) was 30 (11–40) years, and the majority (52.9%) were female. There was no statistical difference between the median age of male and female individuals (*P* = .67), nor age distribution by lineage of individuals with NTV/r RAMs. We identified 4 naturally occurring NTV/r RAMs including T21I, L50F, P108S, and A191V. A191V was the most prevalent (24/34; 70.6%), followed by T21I (7/34; 20.6%). Notably, some strains with T21I also had K90R for the Beta (B.1.351) lineage strains (3/7; 42.9%), and P132H (4/7; 42.9%) for all sequences of the Omicron BE.7 lineage (Table 2).

**Table 1.** Characteristics of Sequences and Metadata With RAMs Included in This Analysis

Total Sequences (n = 5254)		Beta (β) (n = 323; 6.2%)	Delta (Δ) (n = 1314; 25%)	Omicron (o) (n = 3354; 63.8%)	Other (B.1 & C.1.2) (n = 263; 5.0%)
Among 4085 individuals with mutations					
Distribution of sequences with mutations		n = 265	n = 390	n = 3287	n = 143
Proportion (n = 4085; 77.8%), %		6.5	9.5	80.5	3.5
Fraction of sequences with mutations per lineage (%)		265/323 (82.0)	390/1314 (29.7)	3287/3354 (98.0)	143/263 (54.4)
Demographics of individuals with NTV/r RAMs (n = 34)					
Age, median (IQR), y		n = 4	n = 24	n = 6	-
30 (11–40) y		21 (12.5–32.5)	30.5 (8.5–41)	34.5 (24–54)	-
Sex, No. (%)					
Male (n = 16; 47.1%)		2 (50)	11 (45.8)	3 (50)	-
Female (n = 18; 52.9%)		5 (50)	13 (54.2)	3 (50)	-
Year of sampling, No. (%) with NTV/r mutations (n = 34)					
2021 (n = 29; 85.3%)		4	24	1	-
2022 (n = 5; 14.7%)		-	-	5	-
Location of sample collection with major polymorphisms (n = 34)					
Boteti	(n = 2; 5.9%)	2	-	-	-
Chobe	(n = 2; 5.9%)	1	1	-	-
Greater Gaborone	(n = 26; 55.9%)	-	21	5	-
Greater Palapye	(n = 3; 8.8%)	1	2	-	-
Greater Phikwe	(n = 1; 2.9%)	-	-	1	-

Abbreviations: IQR, interquartile range; RAMs, resistance-associated mutations.

**Table 2.** Thirty-four Individuals With NTV/r RAMs

GISAID Accession ID	Sampling Date	Sampling Location	Age, y	Sex	VOC	Pango Lineage	Clade	Mutations in M^PRO^
EPI_ISL_8608080	2021-12-28	Gaborone	24	M	Omicron	B.A.1.1	GRA	P108S, P132H
EPI_ISL_2504107	2021-06-01	Serowe	11	M	Beta	B.1.351	GH	T21I
EPI_ISL_15936262	2022-06-14	Selebi Phikwe	57	F	Omicron	BA.4	GRA	P132H, A191V
EPI_ISL_2648206	2021-05-13	Orapa	37	M	Beta	B.1.351	GH	T21I, K90R
EPI_ISL_2648215	2021-05-14	Orapa	28	F	Beta	B.1.351	GH	T21I, K90R
EPI_ISL_16003679	2022-11-21	Lobatse	38	F	Omicron	BE.7	GRA	T21I, P132H
EPI_ISL_16081968	2022-11-22	Lobatse	31	M	Omicron	BE.7	GRA	T21I, P132H
EPI_ISL_2820316	2021-06-17	Kasane	36	F	Delta	AY.116	GK	A191V, I213V, L227F
EPI_ISL_16553999	2022-11-24	Gaborone	11	M	Omicron	BE.7	GRA	T21I, P132H
EPI_ISL_2820389	2021-06-19	Kasane	14	F	Beta	B.1.351	GH	T21I, K90R
EPI_ISL_3948365	2021-08-12	Molepolole	49	F	Delta	AY.46	GK	L50F
EPI_ISL_3948387	2021-08-16	Gaborone	42	M	Delta	AY.46	GK	A191V
EPI_ISL_3948446	2021-08-26	Molepolole	40	F	Delta	AY.46	GK	A191V
EPI_ISL_3948449	2021-08-16	Bokaa	34	F	Delta	AY.46	GK	A191V
EPI_ISL_3948396	2021-08-10	Gaborone	10	F	Delta	AY.46	GK	A191V
EPI_ISL_3948361	2021-08-12	Gaborone	32	M	Delta	AY.46	GK	A191V
EPI_ISL_3948351	2021-08-24	Gaborone	5	F	Delta	AY.46	GK	A191V
EPI_ISL_3948441	2021-08-24	Gaborone	15	M	Delta	AY.46	GK	A191V
EPI_ISL_3948288	2021-08-24	Gaborone	45	F	Delta	AY.46	GK	A191V
EPI_ISL_3948471	2021-08-23	Lobatse	47	M	Delta	AY.46	GK	A191V
EPI_ISL_3948285	2021-08-23	Lobatse	3	M	Delta	AY.46	GK	A191V
EPI_ISL_3948313	2021-08-26	Molepolole	2	F	Delta	AY.46	GK	A191V
EPI_ISL_4299812	2021-08-31	Kgagodi	29	M	Delta	AY.46	GK	A191V
EPI_ISL_4299801	2021-09-02	Serowe	25	M	Delta	AY.45	GK	A191V
EPI_ISL_5501718	2021-09-07	Gaborone	22	F	Delta	AY.46	GK	A191V
EPI_ISL_5502038	2021-09-15	Gaborone	54	M	Delta	AY.116	GK	A191V, I213V
EPI_ISL_5502181	2022-01-02	Gaborone	54	F	Delta	Omicron	GRA	P108S, P132H
EPI_ISL_5501790	2021-09-27	Gaborone	39	M	Delta	AY.46	GK	A191V
EPI_ISL_5502949	2021-10-11	Tshidilamolomo	13	F	Delta	AY.46	GK	A191V
EPI_ISL_6670243	2021-11-11	Lobatse	34	F	Delta	AY.46	GK	A191V
EPI_ISL_6640868	2021-11-04	Lobatse	7	F	Delta	AY.46	GK	A191V
EPI_ISL_6640899	2021-11-04	Lobatse	7	F	Delta	AY.46	GK	A191V
EPI_ISL_6640869	2021-10-27	Lobatse	7	M	Delta	AY.46	GK	A191V
EPI_ISL_7120921	2021-11-20	Gaborone	48	M	Delta	AY.46	GK	A191V

Abbreviations: M^pro^, SARS-CoV-2 main protein; SARS-CoV-2, severe acute respiratory syndrome coronavirus 2; VOC, SARS-CoV-2 variant of concern.

**Figure 2.**
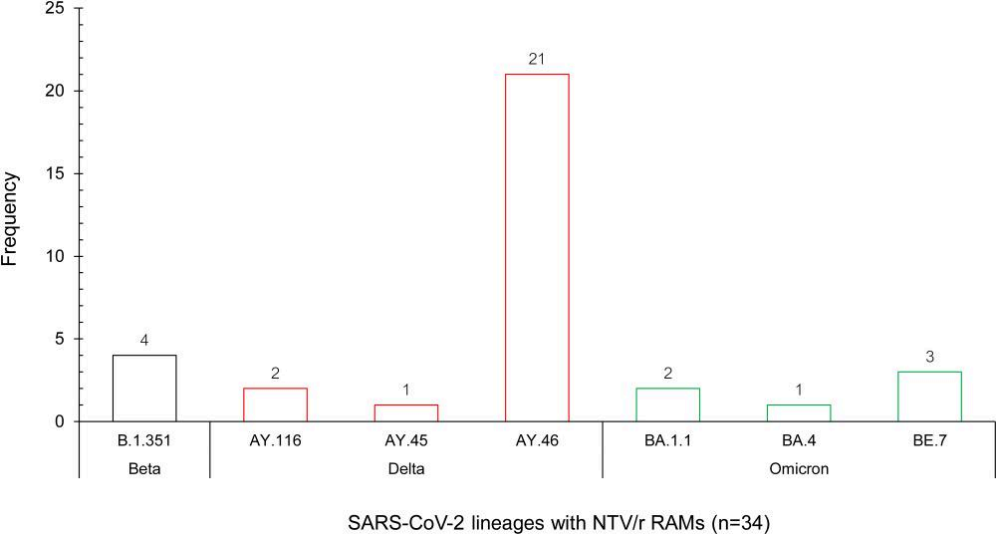
Frequency distribution of NTV/r RAMs stratified by SARS-CoV-2 lineages. Abbreviations: NTV/r, nirmatrelvir-ritonavir; RAMs, resistance-associated mutations; SARS-CoV-2, severe acute respiratory syndrome coronavirus 2.

Other NTV/r RAMs detected in single cases include L50F (1/34; 2.94%) of the Delta (AY.45) VOCs and P108S (2/34; 5.88%) of the Omicron (BA.1.1) VOCs (Table 2, Figure 1B). The RAMs we found confer low-level resistance based on fold reduction in susceptibility to NTV <3 based on the highlighted list of mutations from the 2023 EUA [34]. All the RAMs we report here occurred in SARS-CoV-2 VOCs that circulated in Botswana, including 1.0% (4/403) in the Beta VOCs and 2.2% (24/1107) in Delta VOCs, as opposed to 0.18% in the Omicron VOCs (6/3310). The majority of RAMs (21/34; 61.8%) were identified in Delta sublineage AY.46 (Figure 2).

### Phylogenetic Relatedness of Sequences With Nirmatrelvir-Ritonavir RAMs

**Figure 3.**
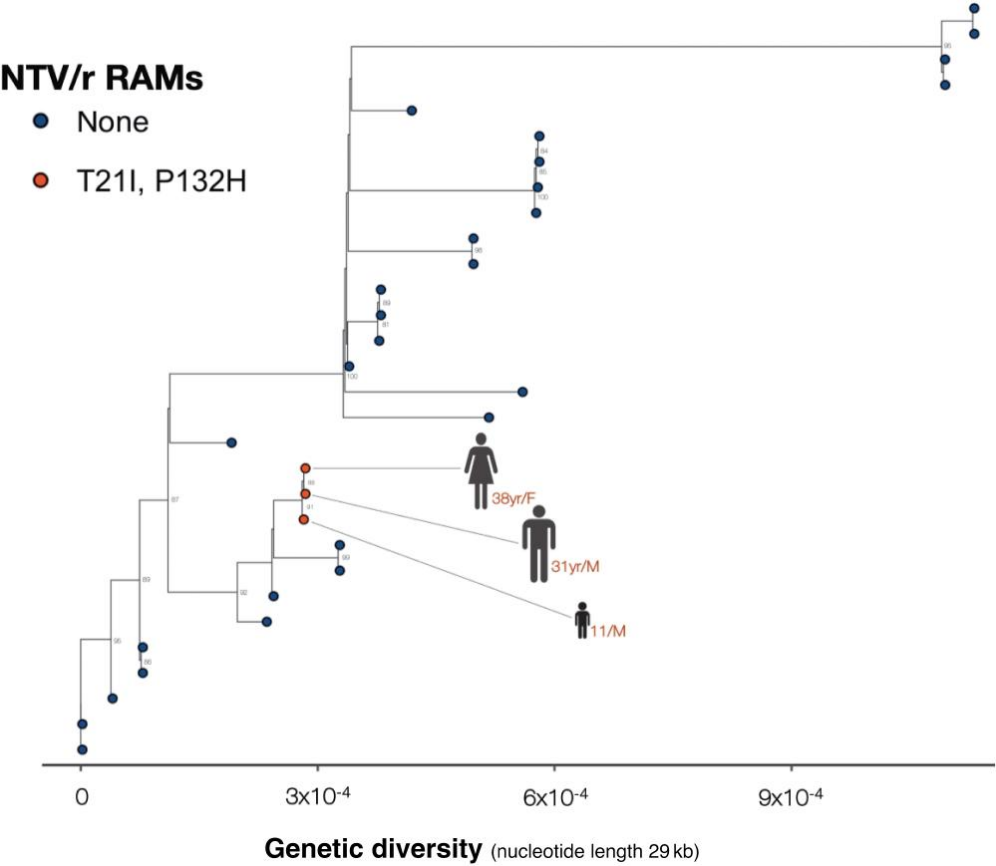
Maximum-likelihood phylogenetic tree comparing all available complete and high-coverage Botswana sequences (n = 30). Samples from the clade containing BE.7, from the branch of Lobatse-Family (red), were sequenced as part of contact tracing.

Among the 6 cases with Omicron VOCs and NTV/r RAMs (Figure 2), 3 who were infected by Omicron BE.7 strains were epidemiologically linked, and a phylogenetic analysis based on ML method close clustering supported this idea, with a posterior probability >90%. This suggests a high likelihood of transmission-based RAMs (Figure 3).

### Diversity of the M^pro^ Gene Among Sequences Isolated in Botswana

**Figure 4.**
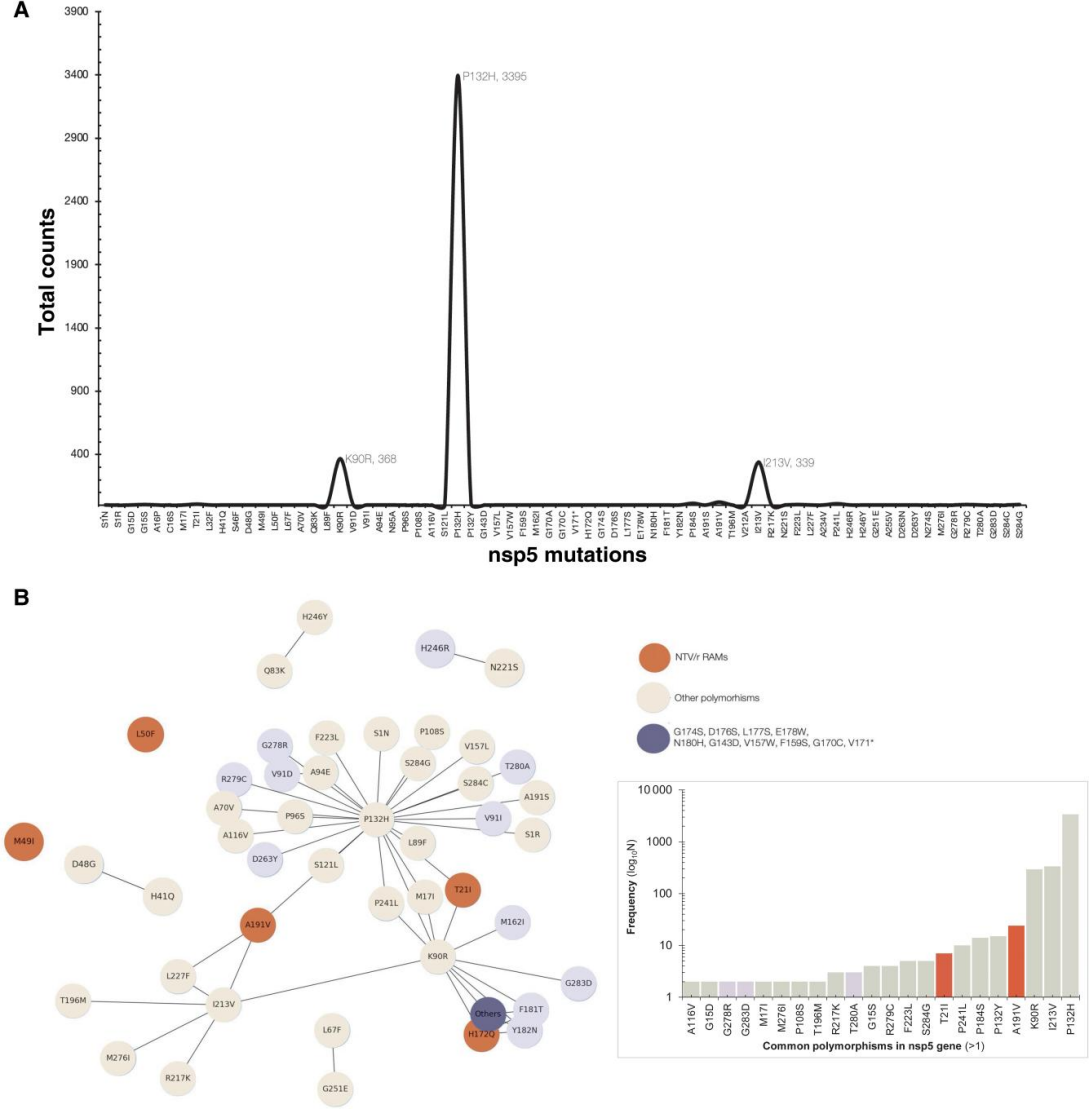
A, Frequency line plot showing the distribution of mutations in the ns5p gene among the 5370 sequences generated in Botswana as of September 30, 2023 (Figure 4B). The network plot summarizes the mutations counts (>1). Each node represents the mutation, and the ones that coexist in the sequences are connected. Bigger nodes indicate a high proportion of sequences and vice versa.

To assess NTV/r RAMs in Botswana, this analysis utilized a reference-based MSA data set encompassing 5254 complete SARS-CoV-2 sequences, totaling 1 427 411 amino acids (a.a), representing both contemporary and earlier circulating viral lineages. Overall, there were 4085 (77.8%) sequences with at least 1 a.a substitution in the M^pro^ gene (Supplementary Table 2). These contained 4169 a.a changes at 68 positions of M^pro^ (306 a.a). Based on these substitutions, the overall diversity index of the MSA was 0.29%. The diversity index was expressed as a total of 4169 a.a substitutions observed over all the 1 427 411 a.a in the MSA, excluding indels. Apart from lineage-defining mutations such as P132H that occurred in almost all the Omicron sequences, we report here that, overall, I213V (n = 335) was the most frequent mutation, followed by K90R (n = 293) (Figure 4A).

Of the 4085 M^pro^ sequences with at least 1 mutation, 1 exhibited 14 mutations, 2 sequences contained 3 mutations each, and 67 sequences had mutations in pairs. The majority, comprising 4015 sequences, featured a single mutation each (Supplementary Table 2). Similarly, among the 70 sequences with at least 2 mutations in the M^pro^ gene, the majority included P132H (n = 44; for Omicron sequences), I213V (n = 10), and/or K90R (n = 9) (Figure 4B). The 68 positions with mutations in the M^pro^ gene were stratified according to their potential impact and thus were NTV/r RAMs (n = 5), putatively deleterious (n = 27), or polymorphisms (n = 36).

### Evaluating the Potential Impact of Uncharacterized Polymorphisms in the M^pro^ Gene

**Figure 5.**
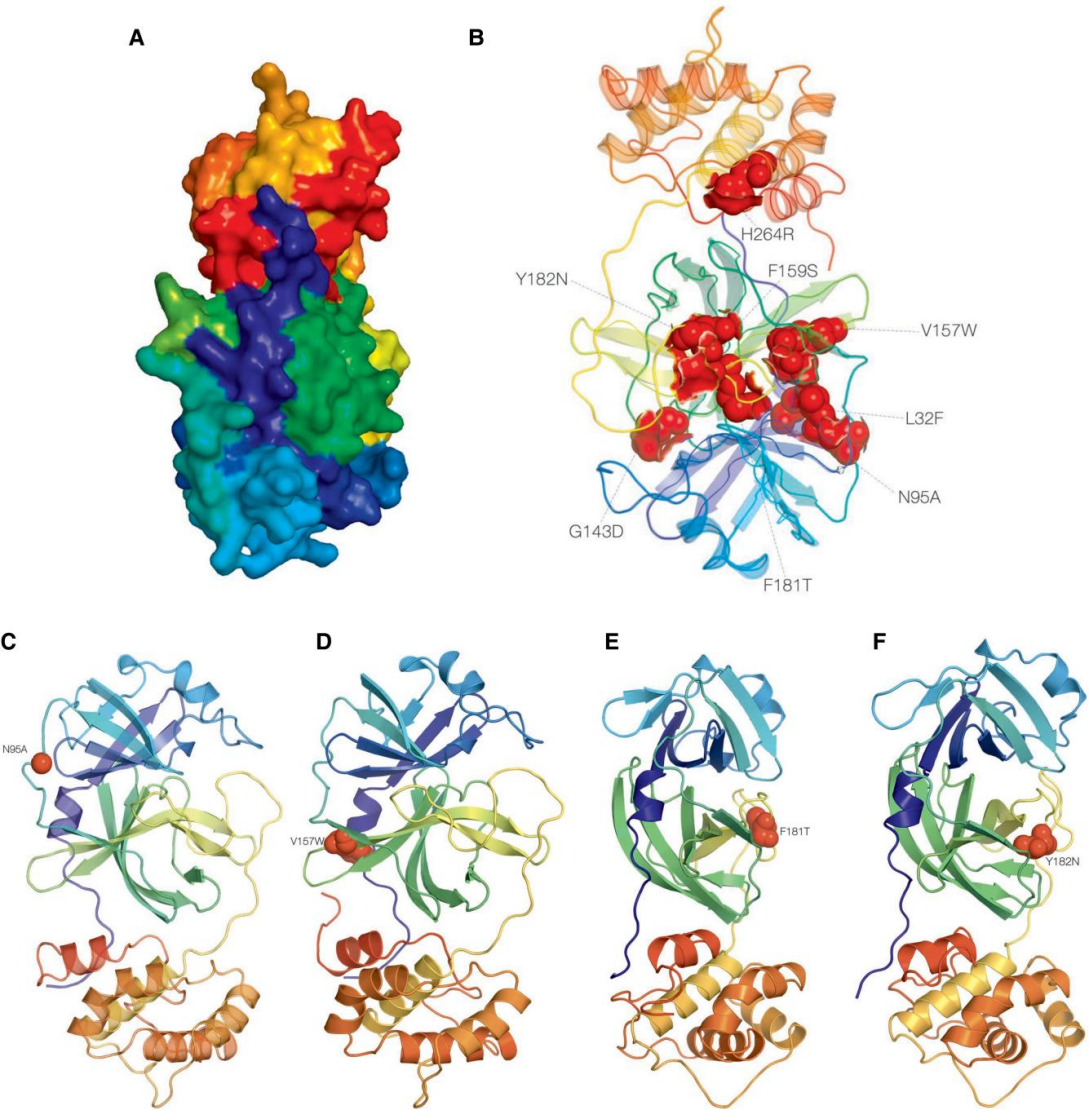
3D structure of ns5p the gene. A, 2duc (http://dx.doi.org/10.2210/pdb2duc/pdb), the crystal structure of SARS coronavirus main proteinase (3CL^pro^); annotated to surface topology using PyMOL 3. A, The Mpro gene is a 306-a.a-long cysteine protease that hydrolyzes viral polyproteins to create functional proteins nsp4–nsp16 in virus replication, which is essential to the virus life cycle [13]. It has an estimated molecular weight of 33 796.64 Da, instability index 27.65, aliphatic index 82.12, and GRAVY score of −0.019 [53]. B, Annotated mutations in 2duc, the crystal structure of SARS coronavirus main proteinase (3CL^pro^). The deleterious mutations have been highlighted using surface and topology and are color-coded in red. C, Annotated putative deleterious mutation N95A in the 2duc, the crystal structure of SARS coronavirus main proteinase (3CL^pro^). D, Annotated putative deleterious mutation V157W in the 2duc, the crystal structure of SARS coronavirus main proteinase (3CL^pro^). E, Annotated putative deleterious mutation F181T in the 2duc, the crystal structure of SARS coronavirus main proteinase (3CL^pro^). F, Annotated putative deleterious mutation Y182N in the 2duc, the crystal structure of SARS coronavirus main proteinase (3CL^pro^). Abbreviations: a.a, amino acid; NTV/r, nirmatrelvir-ritonavir; RAMs, resistance-associated mutations; SARS, severe acute respiratory syndrome.

At least 2 prediction tools detected 27 out of 63 mutations (42.9%) as putatively deleterious and, thus, likely to alter the molecular function of the protein (Table 3). Ten were predicted as putatively deleterious based on PolyPhen [38] and SNAP2 [35, 36], and predictions of 4 mutations (N95A, V157W, F181T, Y182) were rare (Figure 5A–E), each occurring with a prevalence of <0.000001 (1 in a million) in sequences from clinical cases worldwide from GISAID as of November 12, 2023 (Table 3).

**Table 3.** Predicted effects of functionally characterized deleterious mutations of the ns5p region of SARS-CoV-2.

Mutation in M^pro^ gene	SNAP2		PolyPhen (Deleterious effect) Mutation sensitivity (score:1-8) 1=Low; 8=High	Global Frequency as of October 22, 2023
	Score	Expected Accuracy		
C16S	64	80%	7**	68
L32F	11	59%	7**	42
V91D	72	85%	8***	426
V91I	6	53%	5	441
N95A	55	75%	6*	2
G143D	93	95%	7**	146
V157W	56	75%	8***	2
F159S	70	85%	8**	13
M162I	38	66%	8**	7,669
V171D	6	53%	5*	101
G174S	80	91%	4	14
D176S	27	63%	4	179
L177S	61	80%	5*	43
E178W	14	59%	4	46
F181T	63	80%	7**	6
Y182N	93	95%	7**	7
A234V	25	63%	6*	7,279
H246R	20	63%	7**	189
D263Y	7	53%	5	87
G278R	14	59%	5	691
R279C	9	53%	5	4,864
T280A	4	53%	3	540
G283D	90	95%	5	813

*deleterious effect ranked by Phyre2: *deleterious, **more deleterious, ***most deleterious

## DISCUSSION

To our knowledge, this is the first study to examine the baseline prevalence of mutations associated with NTV/r resistance in Botswana. Our results report a low prevalence (0.65%) of naturally occurring NTV/r RAMs among sequences reported in Botswana from all the epidemic waves. NTV/r RAMs were significantly higher (*P* < .001) in Delta (24/35) compared with Beta (4/34) and Omicron (6/34) sequences in Botswana. We did not report any mutations that were associated with high-level resistance toward NTV/r. Our analysis utilized a comprehensive data set that adequately represents all 9 COVID-19 zones (Supplementary b1) in Botswana across all 5 epidemic waves. During the time the samples were collected, NTV/r was not available in Botswana. This may have contributed to the very low prevalence of NTV/r RAMs in Botswana. Although modest, this prevalence is slightly higher compared with 0.5% reported globally and 0.16% reported by a Canadian cohort study [32, 33]. The global analysis of NTV/r RAMs by Ip and colleagues [33] utilized publicly available sequences from GISAID.

In the distribution of the prevalence of NTV/r resistance by continent, South America had the highest resistance (29 032 per million), followed by Africa (24 437 per million) and North America (4809 per million). However, country-specific estimates and patient characteristics were not provided, limiting the contextual interpretation of the results. Our study was population specific, and the NTV/r reference mutations list we used was recently updated.

Among the variants with RAMs, we observed that the majority occurred in the SARS-CoV-2 VOCs—Beta (November 2020 to March 2021), Delta (April to August 2021), and Omicron (November 2021 to date)—circulating in Botswana. Even though Omicron VOCs have predominated the current epi-wave (November 2021 to present) in Botswana and have become the dominant lineage worldwide, it was previously reported that NTV/r has been shown to work against Omicron and its subvariants [11]. Other studies have reported that NTV/r is safe and effective in treating symptomatic COVID-19, reducing the risk of progression to severe infection by 89% compared with placebo [7, 11]. Even though our results reveal that the occurrence of NTV/r resistance is very low in Omicron variants, we recommend that follow-up studies track NTV/r resistance after implementation of NTV/r within our setting. We observed higher rates of NTV/r RAMs among Delta sequences. Further studies are warranted to investigate this trend at the population and global levels.

The accumulation of mutations in SARS-CoV-2 has played a major influence on pathogenesis and clinical outcomes and shaped the COVID-19 pandemic [39, 40]. The most important concern about different SARS-CoV-2 variants is the risky changes that could worsen the severity of the disease and increase transmission [41] or reduce the effects of vaccines and antivirals [42]. Some studies have also indicated that immunocompromised individuals are at increased risk of prolonged SARS-CoV-2 infection [43–46], which can ultimately lead to the accumulation of potentially deleterious mutations identified in silico (eg, NTV/r RAMs) [44]. Even so, NTV/r resistance in cohorts including immunocompromised patients remains understudied. In a recent study by Duan and colleagues, the emergence of NTV/r resistance was associated with persistent infections and/or immune-suppressed individuals [45]. Apart from cases related to possible emergence of RAMs, we observed links between some cases. For instance, the 3 cases of BE.7 were sequences isolated from a single family through contact tracing (Figure 2). Consequently, we emphasize the urgent need for enhanced surveillance of circulating SAR-CoV-2 viral isolates, as strains with RAMs may be potential catalysts for the next pandemic.

In this study, we identified 4 RAMs in the SARS-CoV-2 M^pro^ that confer resistance to the protease inhibitor NTV/r. Based on biochemical assays using recombinant SARS-CoV-2 M^pro^–containing mutations, the 4 SARS-CoV-2 M^pro^ substitutions led to <3-fold reduced nirmatrelvir activity (fold-change based on K_i_ values): 1.6 for T21I, 0.2 for L50F, 2.9 for P108S, and 0.8 for A191V, respectively. This suggests that they confer low-level or an unclear level of resistance to NTV/r [30]. A total of 7 individuals had T21I, which is also associated with decreased antiviral susceptibility [30]. Apart from this study, the preexistence of NTV/r RAMs—T21I, L89F, and L205V—has been reported in low frequencies [32, 33]. In this study, we did not observe any variations at T190, a residue that interacts with NTV/r, or P252 and T304, both of which have been shown to reduce NTV/r activity in vitro [11]. Mutation H41Q, which is a component of the catalytic dyad critical for the protease's function and is involved in drug binding, was observed in 1 sample and was found to cooccur with the polymorphism D48G [31]. We also did not find any mutation at E166, a key residue that interacts with nirmatrelvir, including polar contact with the pyrrolidone group and hydrogen bonds between the tert-butyl moiety of nirmatrelvir [8, 46]. E166 has also been associated with loss of viral fitness and high-level resistance to NTV/r [11, 30]. Similarly, S144E has been shown, in vitro, to confer the greatest reduction in the inhibition of nirmatrelvir on 3CL^pro^ activity [11, 47]. We did not find any mutation changes at S144. In addition to the RAMs we’ve reported, we observed several other mutations that have been previously associated with reduced NTV/r activity but have been excluded in the updated EUA-2023 list [34]; for example, H172Q (n = 1), M49I (n = 1), and G15S/D (n = 6). Mutation M49I has been previously associated with a decrease in the activity of NTV/r without a significant loss of protease activity [30]. Notably, we observed 14—K90R, V157W, H172Q, F181T, Y182N, G143D, F159S, G170C, V171*, G174S, D176S, L177S, E178W, N180H—naturally occurring mutations in the M^pro^ sequence of an unvaccinated, presumed NTV/r-naïve, middle-aged female (47 years; EPI_ISL_1363757). Although we did not find any RAMs, we observed other variations at positions known to confer resistance such as H172. Instead of H172Q, H172Y + P252L in M^pro^ confers high-level resistance (K_i_ values = 180) [34].

Other polymorphisms such as P132H were observed predominantly among the Omicron M^pro^, indicating that this mutation occurs naturally and is not driven by selection pressure from the use of NTV/r. The P132H mutation has been shown to be susceptible to covalent inhibitors such as NTV/r; thus it decreases thermal stability without compromising catalysis or small-molecule drug inhibition [48]. Also, K90R was also observed at high prevalence; however, these amino acid substitutions did not affect drug antiviral activity [49]. Thus, NTV/r is likely to retain its antiviral activity against the emerging Omicron subvariants. However, it is important to continue active surveillance and testing of new variants for drug resistance to enable early identification of drug-resistant strains. We have characterized some mutations using in silico analyses. In silico prediction models have been tested and validated using phenotypic studies in several SARS-CoV-2 mutations including NTV RAMs [26, 51] for resource-limited settings; these approaches provide a faster and more cost-effective way of providing clinically relevant findings. However, there are still limited data on the clinical utility of in silico prediction models for NTV/r RAMs; therefore, follow-up functional studies should be conducted to determine the resistance level conferred by these mutations.

We further assessed the overall diversity of the M^pro^ gene among sequences in Botswana. Overall, we observed low variability (diversity index <1%) across the different VOCs of sequences from Botswana. This corroborates a study that showed that the nonsynonymous variant rate for the protease is >10-fold lower than that for the viral polymerase [50]. A high degree of sequence and structural conservation has been reported in treatment-naïve populations [50]. Consequently, this may explain the relatively low prevalence of resistance to NTV/r in several studies [30]. Even though we report M^pro^ mutations in 5 positions, this study is based on the highlighted list of RAMs provided by the FDA EUA; among the remaining 63 polymorphisms we observed, some could potentially affect the efficacy of NTV/r. Among the VOCs, Beta variants were the most diverse. Given that variants with high frequency (>15%) are likely to represent viral adaptation in the face of selective pressure, prescreening of polymorphisms in the M^pro^ gene is crucial. Here, we further employed our previously reported in silico algorithm to characterize and evaluate the impact of putative deleterious mutations (N95A, V157W, F181T, Y182). Similar approaches of using in silico prediction models to screen putative deleterious mutations have been used to identify RAMs of various antivirals of SARS-CoV-2, of which some candidate mutations have been validated using phenotypic studies [31, 51]. This study is the first to perform in-depth analyses and assess potential impact on several polymorphisms in M^pro^ on protein folding and function. Overall, we observed that the putative deleterious mutations are mostly rare (1 in a million). Nevertheless, further research (functional characterization) is necessary to explore any potential relationship with NTV/r resistance.

To investigate whether strains with RAMs could be transmitted, we employed phylogenetic analysis based on the ML method and 1000 bootstrap values to construct a time-calibrated phylogenetic tree of sequences with NTV/r RAMs of epidemiologically linked cases. The phylogenetic analysis indicated several clusters among sequences supported by posterior probabilities >90%, suggesting a higher likelihood of transmission-based RAMs. This highlights the importance of genomic surveillance, combined with phylogenetic and phylogeography analyses, as a public health intervention tool to elucidate intertransmission dynamics and combat the spread of pathogens such as SARS-CoV-2 [52].

This study had some limitations. The data presented in our study originated from a SARS-CoV-2 routine testing program and were de-identified. This limited our ability to link patients to their clinical histories, including information on HIV status and ART usage. This limited us from determining the prevalence of NTV/r-RAMs by HIV status, which could have provided insight on the impact of immunosuppression on NTV/r resistance. It would have been interesting to assess if any correlation exists between immunosuppression or, especially, ongoing protease inhibitor–based antiretroviral therapy for HIV. However, we anticipated that a substantial proportion (15%–20%) of the samples collected for sequencing originated from people with HIV, as this reflects the prevalence of HIV within the general population of Botswana. One of the main strengths of the study is the ability to characterize NTV/r-DRMs by variants of concern, which revealed that the use of NTV/r among people infected with Delta SARS-CoV-2 could be impacted. Overall, the study findings provide valuable insights on NTV/r resistance in Botswana and surrounding countries in Sub-Saharan Africa and any country that is still reporting SARS-CoV-2 cases driven by Omicron. Our findings emphasize the importance of ongoing surveillance, research, and adoption of the use of SARS-CoV-2 antiviral drugs including Paxlovid to effectively combat the evolving landscape of the COVID-19 pandemic.

## CONCLUSIONS

In our analysis of sequences from routine SARS-CoV-2 national diagnostic testing and sequencing in Botswana, we observed an overall relatively low prevalence of NTV/r RAMs; higher rates were observed in the Beta VOCs and Delta VOCs compared with the Omicron variants (*P* < .001). Among the polymorphisms observed in the M^pro^ gene, we characterized them using in silico approaches and determined specific unreported rare polymorphisms with deleterious effects that may potentially cause resistance to antivirals including NTV/r. Hence, future studies should focus on functional characterization to assess if there is any potential influence on drug resistance. Our findings recommend the potential use of NTV/r in Botswana, especially during the current epidemic wave driven by Omicron VOCs; however, we recommend continued surveillance efforts to monitor for emerging resistance variants as NTV/r use expands.

## Supplementary Material

ofae344_Supplementary_Data

## Supplementary Data

Supplementary materials are available at *Open Forum Infectious Diseases* online. Consisting of data provided by the authors to benefit the reader, the posted materials are not copyedited and are the sole responsibility of the authors, so questions or comments should be addressed to the corresponding author.
