# Correction to: Low Prevalence of Nirmatrelvir-Ritonavir Resistance-Associated Mutations in SARS-CoV-2 Lineages From Botswana

**DOI:** 10.1093/ofid/ofae546

**Published:** 2024-09-30

This is a correction to: Wonderful T Choga, Ontlametse T Bareng, Natasha O Moraka, Dorcas Maruapula, Irene Gobe, Nokuthula S Ndlovu, Boitumelo J L Zuze, Patience C Motshosi, Kedumetse B Seru, Teko Matsuru, Matshwenyego Boitswarelo, Mogomotsi Matshaba, Tendani Gaolathe, Mosepele Mosepele, Joseph Makhema, Trevor J M Tamura, Jonathan Z Li, Roger Shapiro, Shahin Lockman, Simani Gaseitsiwe, Sikhulile Moyo, Low Prevalence of Nirmatrelvir-Ritonavir Resistance-Associated Mutations in SARS-CoV-2 Lineages From Botswana, *Open Forum Infectious Diseases*, Volume 11, Issue 7, July 2024, https://doi.org/10.1093/ofid/ofae344

In the originally published version of the manuscript, the image for Table 2 was duplicated in Table 3. Emendation, the correct image for Table 3, is now made within the article.

