# Supplementary material for: Low Prevalence of Nirmatrelvir-Ritonavir Resistance-Associated Mutations in SARS-CoV-2 Lineages From Botswana
Source: Open Forum Infect Dis. 2024 Jul 2;11(7):ofae344. doi: 10.1093/ofid/ofae344 (PMC11250512; doi:10.1093/ofid/ofae344)
Supplement: ofae344_Supplementary_Data [file ofae344_supplementary_data.zip › Supplementary Table S001.docx]

**Supplementary Table 1.** Mutations in the M^pro^ genes that can confer resistance to Nirmatrelvir.

| SINGLE MUTATION | mutations that CONFER resistance in combination |
| --- | --- |
| A173V | F140L+A173V |
| A191V | H172Y+P252L |
| A193P | L50F+E166V |
| C160F | L50F+F140L+L167F+T304I |
| E166A | L50F+T304I |
| E166V | T135I+T304I |
| F140L | T21I+A173V |
| H172Y | T21I+A173V+T304I |
| L167F | T21I+C160F+A173V+V186A+T304I |
| L50F | T21I+E166V |
| P108S | T21I+L50F+A193P+S301P |
| P252L | T21I+S144A+T304I |
| R188G | T21I+T304I |
| S144A | A173V+T304I |
| S301P | T21I+S144A |
| T135I |  |
| T169I |  |
| T21I |  |
| T304I |  |
| V186A |  |
